# How to address work impairment in patients with disorders of the gut–brain interaction?

**DOI:** 10.1002/ueg2.12429

**Published:** 2023-06-14

**Authors:** Daniel Keszthelyi

**Affiliations:** ^1^ Division of Gastroenterology‐Hepatology Department of Internal Medicine Maastricht University Medical Center+ Maastricht The Netherlands

**Keywords:** disorders of the gut‐brain interaction, functional dyspepsia, irritable bowel syndrome, visceral pain, work impairment

According to the WHO, people are absent from work for 11 days on average per year due to illness.^1^ In the EU27 and Norway, between 3% and 6% of the total workforce is absent at any given point in time, mostly due to health problems.^2^ This costs an estimated 2.5% of the GDP.^2^

Anyone can easily relate to absence from work due to a transient infection such as influenza. A typical full‐time employee could expect to lose about 3½ of their 5 work days in a given week due to absenteeism and presenteeism from an influenza infection.^3^ Fortunately, such absences, although they may occur more than once a year, are generally characterized as reasonably short periods.

Yet there is something less recognizable that substantially affects work productivity: disorders of the gut‐brain interaction (DGBIs), formerly referred to as functional gastrointestinal disorders. This group of disorders includes common conditions such as irritable bowel syndrome (IBS) and functional dyspepsia. In this issue, Frändemark et al.^4^ published a study using data from the Rome Foundation Global Epidemiological Study including 16,820 individuals across 8 countries of whom 7111 with a DGBIs. Their study showed significantly higher work impairment in patients with DGBIs than those without. This difference was even more pronounced when patients had a painful DGBI or a DGBI affecting more than one anatomical region. Fatigue, psychological distress, and somatic symptom severity were also independently associated with work impairment.

These findings are very much in line with previous studies investigating IBS. Three previous cohort studies, from Sweden,^5^ the UK^6^ and the Netherlands,^7^ respectively, all pointed to substantial work impairment (see Table 1). More specifically, Goodoory et al.^6^ found that a mean of 1.97 hours of work per week (equal to 90.5 h per year) is lost due to IBS. In a study performed in a US population, Ballou et al.^8^ showed that IBS symptoms affected work or school productivity for an average of 8 days out of the month and led to absence from work/school for 1.5 days per month because of IBS.

**TABLE 1:** Overview of selected studies investigating work impairment in patients with irritable bowel syndrome (IBS).

Study	Population	Absenteeism	Presenteeism
Frändemark et al.^5^	525 interview‐confirmed Rome III patients (Sweden)	24.3%	86.3%
Goodoory et al.^6^	725 self‐identified Rome IV IBS patients (UK)	28.5%	85.6%
Bosman et al.^7^	419 interview‐confirmed Rome III and IV IBS (Netherlands)	24.1%	34.4%

Any chronic health condition impacts one's life and this extends to their work environment. This is surely true for IBS and other DGBIs. However, the lack of recognition of IBS as a legitimate disorder among the general public, and more specifically employers, contributes to the negative influence of IBS symptoms on job performance. Not being aware of the potential impact of these conditions can put additional strain on employee‐employer relations.

As far as healthcare of IBS patients is concerned, it therefore does not suffice to assume that therapies or interventions aimed at decreasing IBS symptoms in general will necessarily lead to equivalently relevant decreases in work impairment. Healthcare professionals should therefore actively engage in exploring the specific work‐related impact of symptoms. Additional effort from employers and occupational healthcare specialists is also required to ensure necessary adjustments to the working environment and conditions when appropriate, for instance, proper access to clean toilet facilities.

Further, elements of specific work‐related aspects should be integrated into treatment programs for IBS and other DGBIs. For example, it has previously been shown that cognitive behavioral therapy‐based comprehensive self‐management for IBS was superior to usual care in improving overall work productivity loss and activity impairment with sustained effects up to 12 months. Such intervention was found to be particularly beneficial for IBS patients with greater baseline work and activity impairments.^9^ The application of such approaches as part of IBS management strategies will not only benefit patients but also employers in order to reduce indirect costs associated with presenteeism. Let's get to work!

## CONFLICT OF INTEREST STATEMENT

The author has no conflicts of interest to declare.

## Data Availability

Data sharing is not applicable to this article as no new data were created or analyzed in this study.
